# Hemophagocytic lymphohistiocytosis triggered by Hashimoto thyroiditis during pregnancy: a rare case report and diagnostic challenge

**DOI:** 10.1097/MS9.0000000000004171

**Published:** 2025-10-20

**Authors:** Rabia Arshad, Tayyab Husnain, Talha Kashif, Maria Qadri, Hammad Javaid, Anurag Jha, Muhammad Nabeel Saddique, Rana Uzair Ahmad, Muhammad Awais, Ursula Abu Nahla

**Affiliations:** a Mayo Hospital, Lahore; b King Edward Medical University, Lahore, Pakistan; c Jinnah Sindh Medical University, Karachi, Pakistan

**Keywords:** autoimmune disorder, case report, Hashimoto’s thyroiditis, hemophagocytic lymphohistiocytosis, pregnancy

## Abstract

**Background:**

Hemophagocytic lymphohistiocytosis (HLH) is a rare and potentially fatal hyperinflammatory syndrome marked by the excessive activation of macrophages and cytotoxic lymphocytes. Although typically secondary to infections, malignancies, or systemic autoimmune diseases, its association with organ-specific autoimmune conditions such as Hashimoto’s thyroiditis is uncommon, particularly during pregnancy.

**Case presentation:**

A 27-year-old pregnant woman at 22 weeks gestation with a 3.5-year history of Hashimoto’s thyroiditis presented with progressive jaundice, high-grade fever, hepatosplenomegaly, and pancytopenia. Laboratory evaluation revealed hyperbilirubinemia, elevated lactate dehydrogenase (LDH), and hyperferritinemia. Infectious, malignant, and systemic autoimmune causes were excluded. HLH was diagnosed based on HLH-2004 criteria, despite the absence of hemophagocytosis on bone marrow biopsy. She was treated with corticosteroids and cyclosporine. Due to clinical deterioration, a medically supervised delivery was performed at 24 weeks, resulting in neonatal survival and maternal improvement.

**Clinical discussion:**

The diagnosis of HLH in pregnancy is challenging due to overlapping physiological changes. In this case, Hashimoto’s thyroiditis and pregnancy-induced immune modulation may have contributed to immune dysregulation. Early multidisciplinary intervention and immunosuppressive therapy led to favorable outcomes.

**Conclusion:**

This case highlights the need for high clinical suspicion of HLH in pregnant patients with autoimmune conditions and systemic inflammatory symptoms. Prompt recognition, multidisciplinary management, and consideration of early delivery are critical to optimizing maternal and fetal outcomes.

## Introduction

Hemophagocytic lymphohistiocytosis (HLH) is a rare, life-threatening condition of immunological dysregulation characterized by the excessive activation of cytotoxic lymphocytes and macrophages and production of cytokines resulting in systemic inflammation and multiple organ failure^[1]^. It normally presents with fever, cytopenias, hyperferritinemia, and hepatosplenomegaly with elevated liver enzymes and can also lead to widespread organ failure. It can be primary which is familial or secondary which is most triggered by infection, malignancies and other autoimmune disorder^[1,2]^.

**HIGHLIGHTS**

- A rare case of hemophagocytic lymphohistiocytosis (HLH) was triggered by Hashimoto’s thyroiditis during pregnancy.
- Diagnosis was challenging due to overlapping pregnancy symptoms and absence of hemophagocytosis on biopsy.
- Early immunosuppressive therapy and medically supervised delivery led to maternal recovery and neonatal survival.

Hashimoto’s thyroiditis (HT), also known as chronic lymphocytic or autoimmune thyroiditis, is an autoimmune thyroid disorder characterized by increase in thyroid volume and the lymphocytic infiltration of thyroid parenchyma. It is also associated with the presence of antibodies to specific thyroid antigens such as thyroglobulin and thyroid peroxidase. It is often presented with local symptoms including dysphagia, dysphonia and dyspnea and systemic manifestations characteristic of primary hypothyroidism^[3]^.

The direct link between the HLH and HT is not well documented; however, the cytokine profile in HT often shows high levels of several interleukins and chemokines which leads to sustained inflammatory response and immune dysregulation and serve a potential link to HLH^[4,5]^.

Here, we present a rare case of HLH in a young pregnant female patient with a history of HT focusing on the clinical presentation, diagnostic difficulties, and treatment approaches. This case report has been reported following the SCARE criteria for medical case reports^[6]^.

## Case description

### Case presentation

A 27-year-old pregnant female (Gravida 2 Para 0 Aborta 0) with a 3.5-year history of HT, managed with levothyroxine 50 µg/day, presented at 22 weeks of gestation. Her chief complaints included progressive yellowing of the skin and sclera over 2.5 months, high-grade continuous fever ranging 38.5–39.2°C, weight loss, and abdominal pain. Additional findings included oral ulcers, hepatosplenomegaly [liver 21 cm, spleen 13 cm on magnetic resonance imaging (MRI)], lemon-yellow skin discoloration, and patchy hairless areas. Notably, she had received 30 pints of blood transfusions in the preceding 2 months for refractory anemia as packed red blood cells. Her pregnancy had been uneventful until symptom onset, with no nausea, vomiting, diarrhea, rigors, or chills at admission. Laboratory evaluation revealed pancytopenia [Hb 8.9 g/dL (not reported, NR: 12.0–15.5 g/dL], platelets 06 × 10^9^/L (NR: 150–450 × 10^9^/L), WBC 3.0 × 10^9^/L (NR: 4.0–11.0 × 10^9^/L)] (Table1), remarkably increased serum ferritin levels [19 079 ng/mL (NR in females <50 years: 6.24–140.0 ng/mL)], marked liver dysfunction [direct bilirubin 34.80 mg/dL (NR: 0.01–0.30) (Table 2), hypertriglyceridemia 567 mg/dL (NR: <150 mg/dL)], albumin 3.22 g/dL [NR: 3.5–5.0 g/dL]), elevated LDH [909 U/L (NR: 91–245)], and C-reactive proteins (CRP) [18.60 mg/dL (NR: <0.5mg/dL)]. Thyroid function tests indicated suppressed thyroid stimulating hormone (TSH) [0.01 µU/mL (NR: 0.4–4.0 μIU/mL)], low free T3 [1.79 pg/mL (NR: 2.0–4.4 pg/mL)], and normal free T4 [0.83 ng/dL (NR: 0.8–1.8 ng/dL)], with elevated anti-TPO antibodies [1000.0 IU/mL (NR: <35 IU/mL)]. Immunologic workup (serum ANA, anti-dsDNA, anti-Sm, anti-SSA/SSB, anti-Scl70, and anticardiolipin titers) and Coombs tests were negative (Table 3). Infectious etiologies (EBV, CMV, hepatitis B/C, HIV, VDRL, tuberculosis, herpes, and leptospirosis) were also appropriately excluded. Skin biopsy of the patients’ hairless patches was consistent with alopecia areata (AA). Bone marrow aspiration showed predominant hemophagocytic activity. Genetic Testing was not performed due to financial constraints of the patient.

**Table 1:** Sequential hemoglobin levels, platelet counts and WBC counts illustrating hematologic instability and subsequent recovery

Date	Hemoglobin (g/dL)	Platelet count (10^9^/L)	WBC count (10^9^/L)
31 Dec	9.6	4	3.4
01 Jan	8.9	6	3.0
02 Jan	8.3	5	2.7
04 Jan	7.6	4	3.19
12 Jan	6.0	127	8.0
20 Jan	8.2	145	8.4
23 Jan	5.0	111	8.5
24 Jan	5.0	134	8.6
26 Jan	5.2	146	7.8
27 Jan	4.6	160	10.4
01 Feb	8.6	61	8.8
04 Feb	9.0	71	8.6
07 Feb	8.3	102	7.2
08 Feb	10.6	112	8.1
11 Feb	8.2	182	14.0
12 Feb	7.3	158	15.0
13 Feb	10.0	178	14.3
15 Feb	10.1	234	10.8

**Table 2:** Detailed values for ALT, AST, and total bilirubin measured at predefined time points from pre-admission through day 23 of hospitalization

Date (day)	ALT (IU/L)	AST (IU/L)	Bilirubin (direct) (mg/dL)
Preadmission	187	154	34.8
Day 1 (24 Jan)	186	190	14.6
Day 4 (27 Jan)	148	194	11.5
Day 9 (01 Feb)	100	129	16.2
Day 12 (04 Feb)	125	113	17.5
Day 15 (07 Feb)	103	135	13.4
Day 19 (11 Feb)	117	139	9.9
Day 23 (15 Feb)	115	99	10.8

**Table 3:** Diagnostic test results showing autoimmune markers, thyroid function, inflammatory indicators, and hematologic assessment

Test	Result	Reference/cut-off value
Anti-nuclear antibody (ANA)	2.3 (positive)	>1.1 (positive), 0.9–1.1 (borderline), <0.9 (negative)
Anti Sm antibodies	Negative	
Anti RNP antibodies	Negative	
Anti SSA (Ro) antibodies	Negative	
Anti SSB (La) antibodies	Weak positive	
Anti Jo 1 antibodies	Negative	
Anti Scl 70 antibodies	Negative	
TSH	0.01 (low)	0.34–5.60 uIU/ml
Free T3	1.79 (low)	2.5–3.9 pg/ml
Free T4	0.83 (normal)	0.61–1.12 ng/dl
Thyroid peroxidase antibodies (Anti-TPO)	1000 (positive)	<30 (negative), ≥30 (positive)
Direct Coombs test	Negative	
Indirect Coombs test	Negative	
Anti-nuclear antibody (ANA) quantitative	29.0 (negative)	<40 (negative), ≥40 (positive)
Anti HEV IgM	Negative	<0.150 (negative), ≥0.150 (positive)
CRP latex (quantitative)	18.60 mg/L	Less than 5.00 mg
Lactate level	22.00 mg/dL	4.50–19.00 mg/dL
LDH (serum)	1019 U/L	135–214.0 U/L

Empirical antibiotics and prednisone (1 mg/kg/day, 50 mg divided twice daily) were initiated on admission to the hospital. Cyclosporine (50 mg/day) was added after 5 days. Before starting cyclosporine, informed consent was taken and risks and benefits were discussed with the patient. Levothyroxine was continued (50 μg/day orally), and deferoxamine (900 mg/day subcutaneously, 5 days/week) was administered for transfusion-associated iron overload.

Due to fetal risks and maternal instability, a medically supervised vaginal delivery was performed at 24 weeks gestation due to concerns of bleeding while having a cesarean section (C-section), resulting in a neonate with an Apgar score of 4 at 1 minute and 7 at 5 minutes, with subsequent stabilization in the neonatal intensive care unit (NICU), and a birth weight of 480 g. Immediate resuscitation was required with positive pressure ventilation, intubation and surfactant administration, followed by admission to the neonatal intensive care unit (NICU). Nutritional support was initiated with parenteral nutrition, gradually transitioned to enteral feeds. The clinical course was complicated by anemia requiring transfusions, apnea of prematurity, all of which were managed according to NICU protocols.

Post-delivery, the patients’ anemia improved with transfusion (Hb increased from 4 to 10 g/dL by day 10). Her neonate remained in the NICU 4 weeks post-delivery. Eventually after stabilization of both the mother and the baby, they were discharged along with plans for ongoing multidisciplinary follow-up. However, the patient did not return for follow-up.

## Discussion

Our patient a 27-year-old pregnant female with a 3.5-year history of HT presented with progressive jaundice, high-grade fever, weight loss, and hepatosplenomegaly. Her weight was 59 kg at the time of the presentation. Laboratory findings were consistent with HLH revealing pancytopenia, significant hepatic dysfunction with elevated bilirubin and LDH and marked hyperferritinemia. Patient’s clinical picture was further complicated by the fact that she had received 30 pints of blood transfusions for refractory anemia in the preceding 2 months. The diagnosis of HLH was confirmed on the basis of HLH-2004 criteria. She was managed with corticosteroids, cyclosporine, and supportive care leading to significant clinical improvements.

The presented case shares several features with previously reported cases of HLH in autoimmune diseases such as the presence of fever, cytopenias, and hyperferritinemia as seen in SLE associated HLH^[7,8]^. However, our patient’s presentation was unique due to the absence of SLE or other systemic autoimmune diseases with HT and AA being the only autoimmune conditions present. The presence of these organ specific autoimmune diseases indicates that HLH can arise from immune dysregulations other than systemic autoimmune disorders, likely due to shared mechanisms like cytokine storms and impaired T-cell function^[9]^.

Moreover, AA is an autoimmune disorder that leads to non-scarring hair loss as a result of the immune system attacking hair follicles. This condition is often linked to a wider range of autoimmune issues, especially thyroid conditions like HT^[10]^. While the precise mechanisms connecting AA to HT remain unclear, there are shared genetic and immunological pathways between the two conditions. These include disruptions in immune privilege and T-cell-driven inflammation, where CD8^+^ T lymphocytes are crucial in the follicular damage seen in AA, along with similar patterns of inflammatory cell infiltration noted in HT^[11]^. Furthermore, individuals with AA show an increased frequency of thyroid autoantibodies (such as thyroperoxidase antibodies) and related thyroid issues, with hypothyroidism being the most frequently observed thyroid condition in this group^[12,13]^. Even though there are these connections, the existing literature has not investigated the relationship between AA and HLH. Nevertheless, as both HLH and AA involve irregular immune activation, additional studies into the possible connections in severe autoimmune manifestations are necessary^[10,11]^.Furthermore, adding to the diverse clinical manifestations of this condition was the marked hepatic dysfunction observed in our patient which happens to be a rare initial presentation of the condition possibly lead to complexity and delays in diagnosis^[14]^. The presence of AA as confirmed by skin biopsy further adds to the complexity of the case as it may reflect a broader autoimmune involvement in the development of HLH^[7]^.

Additionally, the suggested mechanism of a “Th2 shift” leading to HLH lacks direct support in the search results, as HLH is mainly characterized by a cytokine storm that is dominated by Th1-type cytokines like IFN-γ, with IL-10 being a significant counter-regulatory Th2 cytokine^[15]^. Additionally, the possible link between thyroid dysfunction (such as TSH suppression or reduced T3) and HLH has not been investigated in the existing literature, even though the immunomodulatory effects of thyroid hormones on the immune system are recognized. The search results do not contain specific information regarding thyroid hormone levels or the balance of Th1/Th2 in patients with HLH, revealing substantial knowledge gaps in these topics^[16]^.

Additionally, the diagnosis of HLH in our patient was particularly challenging due to the overlap of symptoms with other conditions such as viral hepatitis, autoimmune hepatitis and lymphoma. Primary liver disorder was suspected initially due to the presence of jaundice and elevated liver enzymes but the rapid progression of symptoms complemented by the absence of any kind of viral markers indicated a systemic inflammatory process^[14]^. However, diagnostic process was further complicated due to pregnancy, as physiological changes such as increased cytokine production, infections and altered immune tolerance can possibly mimic or exacerbate the features of HLH^[17]^. The standard HLH-2004 criteria were significant in guiding the diagnosis of our case^[18]^. Thus, clinicians should rely on a combination of both clinical and laboratory findings to confirm the case of HLH, particularly when all diagnostic criteria are not met.

We found that during pregnancy there is a state of altered immune regulation characterized by a specific shift towards a Th2 dominant cytokine profile possibly for maintaining fetal tolerance^[19]^. This state of immune shift can potentially predispose pregnant females to immune dysregulation, as seen in our patient where the hyperinflammatory state of HLH may have been triggered or exacerbated by pregnancy. The management of HLH in pregnancy is extremely difficult, as there is a need for aggressive immunosuppressive therapy that needs to be balanced against the elevated risk of fetal health deterioration. In our case, the decision to proceed with a medically supervised delivery at 24 weeks gestation was driven by the severity of maternal illness and the need to optimize both maternal and fetal well-being. Our approach of early delivery aligns with other reports of HLH in pregnancy where early delivery has been used to decrease maternal risks and improve outcomes. However, it is important to note that early delivery carries its own risks including increased neonatal mortality and further research is needed to optimize the timing and management of delivery in such cases^[20]^.

Furthermore, the management of HLH in our patient involved a combination of corticosteroids, cyclosporine, and supportive care. Corticosteroids were initiated to suppress the hyperinflammatory response by inhibiting cytokine production and T-cell activation, while cyclosporine was added to target the underlying immune dysregulation by inhibiting calcineurin and reducing T-cell proliferation. This approach is consistent with the HLH-2004 protocol which recommends immunosuppressive therapy for secondary HLH^[18]^. Although the use of cyclosporine in pregnancy is debatable, the decision to use cyclosporine in our patient was based on its favorable risk to benefit profile as compared to other immunosuppressive agents like etoposide in secondary HLH^[21]^. Levothyroxine and deferoxamine were given adjacently to address the accompanying conditions of HT and transfusion related iron overload.

Delayed diagnosis in HLH cases is usually associated with irreversible multi-organ failure and high mortality, early recognition and treatment thus becomes highly critical in this complicated disorder for improving outcomes^[20]^. In our case, the initiation of corticosteroids and cyclosporine led to significant clinical improvement highlighting the importance of timely intervention. Moreover, the patient’s prednisone dose was calculated at 1 mg/kg based on her weight of 59 kg, resulting in 60 mg daily to rapidly control inflammation, while the low cyclosporine dose (50 mg/day or approximately 0.8 mg/kg) was specifically chosen due to pregnancy concerns, prioritizing fetal safety by minimizing potential nephrotoxicity and hypertension risks despite the need for higher immunosuppression^[22,23]^. Deferoxamine was administered at 30 mg/kg/day intravenously for 14 days to address iron overload from recurrent transfusions, with rigorous monitoring including daily auditory testing and weekly ophthalmologic exams to detect early signs of neurotoxicity and retinal damage, given the increased risk of these side effects during prolonged use^[22,23]^.

Our case highlights the importance of timely recognition of HLH in autoimmune patients particularly during pregnancy where physiological changes can lead to misdiagnosis and worsen the symptoms of this highly fatal condition^[20]^. A multidisciplinary team of rheumatologists, hematologists, obstetricians and neonatologists was involved in the management of this case balancing the aggressive immunosuppression with fetal safety ultimately deciding on the early delivery and ensuring the preterm infant’s survival.

One limitation of our case is the lack of genetic testing to rule out primary HLH. While the patient’s history and clinical presentation were more consistent with secondary HLH genetic testing could have provided additional insights into the underlying etiology. Further cases should consider genetic testing to better understand the role of inherited mutations in the development of HLH in patients with atypical presentations^[24]^. Additionally, the development of a standard pregnancy-specific guideline for the management of HLH would greatly aid clinicians in complex scenarios like the one presented here.

## Conclusion

HLH should be taken into account in pregnant patients who have autoimmune diseases, necessitating a collaborative strategy for timely intervention. Future research must establish guidelines for pregnancy and investigate genetic risks to enhance outcomes.

## Data Availability

None.

## Research registration unique identifying number (UIN)

Not required as this is a case report and we have full consent signed from the patient.
